# Rapid *in vivo *Taxotere quantitative chemosensitivity response by 4.23 Tesla sodium MRI and histo-immunostaining features in N-Methyl-N-Nitrosourea induced breast tumors in rats

**DOI:** 10.1186/1475-2867-5-26

**Published:** 2005-08-03

**Authors:** Rakesh Sharma, Richard P Kline, Ed X Wu, Jose K Katz

**Affiliations:** 1 Department of Medicine, W168th Street, Columbia University, New York, NY 10032 USA; 2 Department of Radiology, W168th Street, Columbia University, New York, NY 10032 USA

**Keywords:** Apoptosis, antineoplastics, non-invasive, therapeutic efficacy, Taxotere

## Abstract

**Background:**

Sodium weighted images can indicate sodium signal intensities from different features in the tumor before and 24 hours following administration of Taxotere.

**Aim:**

To evaluate the association of in vivo intracellular sodium magnetic resonance image intensities with immuno-biomarkers and histopathological features to monitor the early tumor response to Taxotere chemotherapy in Methyl-Nitroso-Urea induced rat xenograft breast tumors.

**Methods and Materials:**

Methyl-Nitroso-Urea (MNU) induced rat xenograft breast tumors were imaged for sodium MRI and compared with tumor histology, immunostaining after 24 hours chemotherapy.

**Results:**

Sodium MRI signal intensities represented sodium concentrations. Excised tumor histological sections showed different in vitro histological end points i.e. single strand DNA content of cell nuclei during cell cycle (G1/S-G2/M), distinct S or M histograms (Feulgen labeling to nuclear DNA content by CAS 200), mitotic figures and apoptosis at different locations of breast tumors. Necrosis and cystic fluid appeared gray on intracellular (IC) sodium images while apoptosis rich regions appeared brighter on IC sodium images. After 24 hours Taxotere-treated tumors showed lower 'IC/EC ratio' of viable cells (65–76%) with higher mitotic index; apoptotic tumor cells at high risk due to cytotoxicity (>70% with high apoptotic index); reduced proliferation index (270 vs 120 per high power field) associated with enhanced IC sodium in vivo MR image intensities and decreased tumor size (3%; p < 0.001; n = 16) than that of pre-treated tumors. IC-Na MR signal intensities possibly indicated Taxotere chemosensitivity response *in vivo *associated with apoptosis and different pre-malignant features within 24 hours of exposure of cancer cells to anti-neoplastic Taxotere drug.

**Conclusion:**

Sodium MRI imaging may be used as in vivo rapid drug monitoring method to evaluate Taxotere chemosensitivity response associated with neoplasia, apoptosis and tumor histology features.

## Introduction

In tissue, sodium exists as total extracellular sodium (EC-Na) ~ 139 mM occupying ~0.15 extracellular water spaces and bound intracellular sodium (IC-Na) ~ 15 mM concentrations. The sodium nuclei exhibit slower and faster relaxation times respectively [1]. Tissue sodium concentrations may change during early tumor stages without membrane damage. Heterogeneous tumors showed the enhanced sodium MR image signal and sodium concentration in tumors [2,3]. Increased intracellular sodium along with histopathology and apoptosis related cytomorphological changes can provide the real time information of antineoplastic effects to optimize chemotherapeutic efficacy at the onset [2,4,5].

Docetaxal has gained attention for its cytotoxic effect in cancer prevention. Rapid time-dependent monitoring of docetaxal chemosensitivity by sodium MRI is emerging as clinical tool in cancer therapy [2,5].

Recently, MNU induced breast tumors in rats showed distinct pre-malignant stages such as lymph node invasion, ductal carcinoma, cell proliferation, apoptosis, cyclin D1/p27 expression, codon 12 mutation frequencies in Ha-ras, microsatellite instability and apoptosis [6]. Cytopathological response and in vivo sodium MRI measured the chemosensitivity of primary tumor to drug as first estimate of neoplasm and metastatic sensitivity [7].

For *in vivo *sodium imaging, 4.23 T clinical MRI was developed and evaluated to achieve high resolution of animal tumor and drug monitoring in our laboratory [5]. The present study reports the calibration of the *in vivo *sodium MRI for both EC-Na and IC-Na sodium signal intensities of phantoms consisting varying concentrations of free sodium and bound NaCl dissolved in agarose gel. The novelty of high resolution *in vivo *sodium MRI images with high signal-noise-ratio was the choice of using inversion recovery pulse sequence to measure IC-Na measurement in tumor; and use of single quantum filter for tumor size measurement to compare with histological features, immunostaining maps of tumor to suggest active apoptosis and neoplasia with different tumor stages before and 24 hours post-injection of Taxotere in MNU induced rat xenograft breast tumors. Primary hypothesis was that IC-Na sodium in tumors was enhanced after MNU induced cell destruction and associated with apoptosis. Secondary hypothesis was that Taxotere chemosensitivity effect can be quantitated by biomarkers using sodium imaging, histopathology and histo-immunostaining features. To accomplish it, sodium MRI signal intensities (as rapid quantitative method) were compared with different *in vitro *staining end points to assess tumor response to Taxotere therapy. These results may have implications on utility of *in vivo *drug monitoring by sodium MRI in human breast tumors.

**Figure 1:**
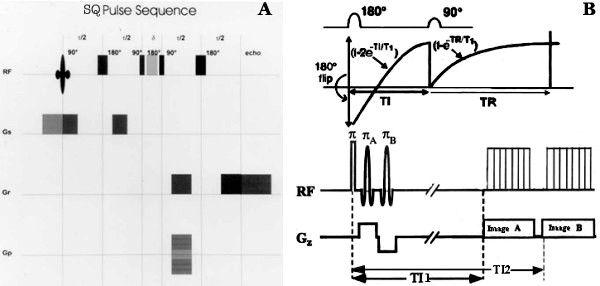
The figure shows a spin echo pulse sequence with single quantum mode (A on left) and inversion recovery pulse sequence (B on right) using two inversion pulses for sodium imaging. The exponential return of the Mz after inversion is shown in graph in the center in relation to the RF pulses. The FID signal, produced by the 90° pulse after TE, has initial amplitude related to the value of Mz at the end of interval TI1 and TI2 due to applied selective π pulse. The insert highlights the unique optimum TI value in distinguishing tumor density associated with IC sodium in tumor.

Sodium MRI depends on sodium nuclei having 3/2 spin states and a quadruple moment. Sodium nuclei exhibit four transitions and two different longitudinal and transverse relaxation constants, a long T2 = 16–30 ms and a short T2 = 0.7–3.0 ms. Sodium MRI utilizes the multiple quantum Na-MRI in a single quantum (SQ) framework as shown in Figure 1 (left panel). Single quantum sodium MRI acquires EC-Na sodium signal-to-noise ratio quickly without the need of paramagnetic shift reagents. We believe that quantum filter interactions may display their effects on nuclear spin transitions as multiple transverse relaxation constants and characteristic IC-Na signal of sodium nuclei in heterogeneous tumor. IC-Na sodium content can be measured by multiple quantum filter (MQF) using shift reagents. Other way is triple quantum filter method that takes long acquisition time. In tumors, sodium nuclei of the outer cell membrane surfaces display an extracellular MQ sodium signal. We propose a rapid and reliable approach of inversion recovery pulse sequence in spin echo sequence to acquire IC-Na sodium high resolution images as shown in Figure 1 (right panel) and previously described elsewhere [8].

## Materials and Methods

Experimental MNU induced Sprague Dawley rat breast tumor model was used as described elsewhere [4]. Breast tumors were imaged *in vivo *using intracellular sodium weighted MRI approach both at baseline and following administration of Taxotere. All rats were used in accordance with animal care IACUC and Columbia University rules for humane treatment of animals. Subsequently, tumors were excised and *in vitro *cell DNA phase staging, single strand-DNA antibody (SS-DNA) staining and histopathological features were measured [9-11]. Tumor areas were delineated on SQ and IR sodium MR images using Optimas 6.5 software and their intracellular sodium was measured by relative signal intensity of known sodium concentration i.e. 1 M in 4% agarose phantom for determination of intracellular [Na]_i_.

### Validation of intracellular and extracellular sodium Magnetic Resonance Imaging of phantoms *in vivo*

Two reference phantoms containing 0.1 M NaCl and 10 mM NaCl in 4% agarose-Na were glued to a plastic animal platform. The 4% agarose-Na phantom was the brightest phantom on the IR image and the NaCl phantom was brightest on the SQ image. For image quality purposes, IR images and SQ images were normalized to the brightest phantom within each slice for comparison using NIH Image J software.

For sensitivity of intracellular sodium MR image intensity, a series of intracellular sodium phantoms was created by dissolving NaCl into four concentrations of agarose (10, 20, 30, and 40% w/v in saline water) for six different NaCl concentrations (5, 20, 50, 100, 120, 150 mM). KCl was added with NaCl to maintain a constant cationic molarity of 150 mM and constant response to radiofrequency coil. The samples were kept at a constant temperature (25°C) and pH (3.54).

Sodium concentrations in tumors were measured by relative sodium concentrations of phantoms placed next to tumor in animals. Mean sodium signal intensity I_k _and sodium concentration per kilogram wet weight in region of interest can be represented as:

*I*_k _= *A*[1 - *B *exp(-TR/T1)]     (1)



where a and b are constants and depend upon signal intensities I_1 _and I_2 _and sodium concentrations C_1 _and C_2 _in both intracellar and extracellular sodium phantoms as following:





where R_1 _and R_2 _are sensitivity factors. SF_1 _and SF_2 _are saturation factors [2].

### Methyl-Nitrosourea (MNU) induced breast tumor propagation

To propagate the tumor in rats, 42 to 50 days old 30 Sprague Dawley rats were obtained from Harlem Sprague-Dawley (Indianapolis, IN) and the 15 rats were injected i.p. with N-methyl-N-Nitrosourea (MNU), at a dose of 50 mg/kg body wt., purchased from (Ash Stevens Inc., Detroit). After injection, the animals were weekly monitored for tumor propagation and tumor development. First palpable tumor was observed and recorded. The animals were fed 4% Purina Chow diet ad libitum and free access to water. The lesions varied in the 0.2 – 1.0 cm range (stage I), presence of cyst, scar, or solid, benign tumor or lesions in the range of 1.0–3.0 cm. (stage II), and malignant or lesions more than 3.0 cm (stage III) [4]. For comparison, 2 animals were injected placebo water in similar way instead of MNU at sham site. No injection was given to 3 animals and these served as control.

### Administration of Chemotherapy

Taxotere (40 mg/ml) was dissolved in the 20% ethanol diluent to a working solution concentration of 6 mg/ml. Taxotere dose levels were 6.0 mg/kg body weight. For it, Taxotere was dissolved in supplied diluent to prepare working solution 100–150 μL and slowly injected into a femoral vein of rats anesthetized by intradermal injection of 1.25 cc of a mixture (Ketamine 10 mg/ml + Xylazine 0.5 mg/ml) at the rate of 1.25 ml/hr (15 mg/hr Ketamine; 0.75 mg/hr Xylazine). Each animal was injected 6 mg/ml Taxotere (i.e. ~1.5 mg/250 gram body weight) calculated from standard equations which convert weight to surface area for small mammals [5].

### Chemosensitive effect of Taxotere on breast tumor

When the tumors were developed approximately 2.0 – 2.5 cm in diameter, the tumor bearing, sham and control animals were treated with Taxotere and sodium imaging was done and repeated following 24 hours after injection. For MRI imaging, animals were anesthetized as mentioned above. In units of animal weight, it was a loading dose of 37.5 mg/kg wt for Ketamine and 1.875 mg/kg for Xylazine. This i.p. injecting dose was sufficient to maintain anesthesia in animals for 1 hour.

### Magnetic resonance imaging and analysis

All imaging experiments were performed on a high field (4.23 Tesla) whole body clinical MRI system at the Columbia University Hatch NMR Center. A small quadrature birdcage radiofrequency coil (50 mm internal diameter, Morris Inc.) and a high strength gradient insert coil (30 mT/m, Bruker model G-33) were employed in this study to achieve 0.5 × 0.5 × 1.0 cubic mm spatial resolution and 1.0 mm in plane resolution.

The anesthetized rats were aligned in a deep groove with the tumor positioned through a small elliptical opening in the plastic holder, thus maintaining the same relative rat eye position to the phantoms from experiment to experiment. 24 slices were acquired for 3D gradient-echo single quantum image (acquisition time 15 min) and subsequently, an inversion recovery image (acquisition time 45 min). Acquisition parameters were: TR = 100 msec, TE = 5.6 ms, FOV = 40 mm, slice thickness = 2.5 mm, inversion time = 25 msec, and flip angle = 90°, acquisition matrix was 64 × 64 × 8. Both inversion and excitation pulses were non-selective pulses [5].

**Figure 2:**
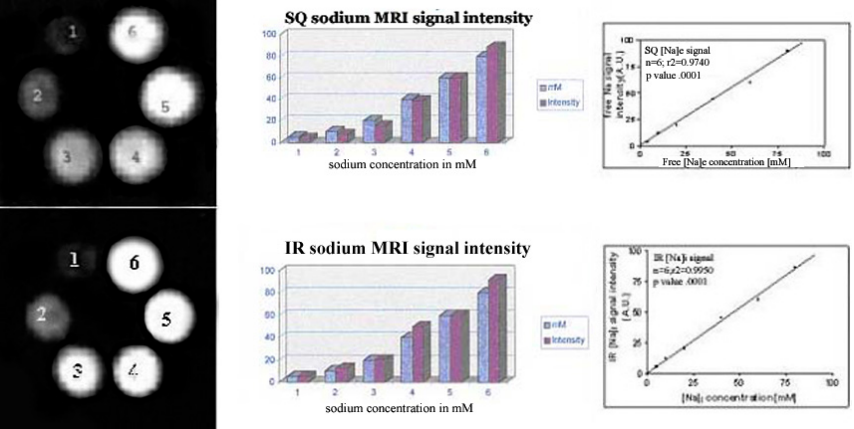
Different 6 concentrations of Na (mM) and their SQ MRI signal intensities are shown in images (left panel) with their relationship as histogram bars and graph (right panels) between sodium concentrations of NaCl solutions and SQ MRI intensities. In second row at bottom, different sodium concentrations and their visibility is shown on left. Histogram bars and graph shows IR sodium MRI signal intensities with different sodium concentrations in 4% agarose.

Analysis of tumor sodium MRI signal intensity change and sodium concentration in all pre- and post- drug treated tumors were measured (in millimoles) relative with corresponding sodium signal intensity in phantom image at its brightest region using NIH J software (see Figure 2). The software puts high quality sodium MRI image sets into common geometrical frame by rotate-translate transform to define region of interest.

### High resolution images with high sensitivity

Minimum duration of 90 degree Rf pulse with minimum saturation time of preamplifier was used to minimize magnetic inhomogeneity and FID. The image signal intensity depends on both Rf receiver and transmitter coils as:

I_k_= R_k_.M_0k_.|sin (Φ_k_)| (5)

where I_k _is signal intensity of k^th ^pixel, R_k _is sensitivity at the coil center, M_0k _is the equilibrium magnetization. The flip angle Φ_k _is function of transmitter gain setting TG as:

Φ_k _= (C.R_k_).10^TG/20 ^(6)

(C.R_k_) represents relative sensitivity R_k _for the k^th ^pixel. The constant C is independent of spatial position. The receiver sensitivity is directly proportional to transmit field distribution for the coil.

### Post-imaging tumor histo-immunostaining end points

The entire neoplastic explant tumor was removed and sliced into 2 mm thick serial segments starting from the cranial aspect marked by suture and proceeding caudally. A touch preparation of the caudal aspect of each slide was prepared and slides were air dried and fixed with 10% phosphate buffered formalin for Feulgen staining to determine nuclear DNA content. Thereafter slices were fixed by immersion in 10% buffered formalin and embedded in paraffin. For histology, 4 micrometer thick sections were prepared of each segment. The MRI co-registered histology sections were stained for hematoxylin and Eosin (H & E) to determine cytomorphology. Different tumor cytomorphic features were counted as number of micrometer squares per mm^2 ^under high power field (HPF) at magnification (× 400) microscope equipped with micrometer squares based on: mitotic rate (number of mitotic figures in micrometer) as mitotic index (MI), amount of apoptosis (20–40 apoptotic nuclei in micrometer square) as apoptotic index (AI), viability (<60% viable cells in micrometer square), cyst space (<100 μm size) and necrosis (<25% necrosis cells in micrometer square) in double-blind manner. The distribution of % extracellular (EC) and intracellular (IC) space was determined with the trichrome stain, where EC appeared light and IC as darker. Malignant cells were characterized according to criteria described elsewhere [4].

### Cell cycle analysis

The amount of s-DNA content was determined morphometrically using the Feulgen stain to measure the amount of s-DNA per cell nucleus stained using a computer aided image analysis (CAS 200) [11]. The S phase was defined as cells with s-DNA content immediate between diploid and tetraploid. On an average, 200 cancer cells were evaluated and their s-DNA content was plotted in histogram form. The histograms were compared to a standard histogram from non-neoplastic cells of the same tumor specimen to determine the standard DNA content for diploid cells (G0/G1) phase of cell cycle and the range of s-DNA content for tetraploid (G2 phase) cells. The proliferation index was expressed as the number of cancer cells in S/G2 phase vs total number of cells (G0/G1 + S/G2) per HPF for each tumor.

### Immunostaining and TUNEL labeling

The immunoperoxidase enzyme staining technique was used to demonstrate proliferating cells by labeling with KI 67 antigen. Formalin-fixed paraffin-embedded sections were processed for heat retrieval for the KI-67 antigen and then incubated with the primary anti-KI 67 antibody. This antibody was linked to a secondary biotinylated antibody. The binding of the target antigen was demonstrated with avidin-biotin-peroxidase technique using 3, 3' diaminobenzidine as substrate for the peroxidase. It generates a brown reaction product in proliferating nuclei. The non-proliferating nuclei was counterstained green (Methyl-green) and the proliferation index (PI) was evaluated using colorimetric difference on computer aided analysis (CAS 200) as reported earlier [12].

### FACS and apoptosis index

The apoptotic nuclei were visualized on formalin-fixed, paraffin-embedded sections using the antibody MAB3299 single-strand DNA(ss-DNA MAb) using immunogen F7–26 and peroxidase conjugated isotype IgM by Flourescence Activated Cell Sorter (FACS) analysis (Chemicon International Inc. Temecula, CA)[13]. The formalin-fixed frozen sections were treated with increasing ethanol dilutions. These sections were treated with 0.1 mg/ml saponin-PBS, Protinase K (20 μg/ml PBS) for 20 minutes. Later slices were washed thrice with distilled water and 50% formamide at 55°C. Soon after slices were quenched with endogenous peroxidase (in 3% hydrogen peroxide for 5 minutes) and treated with 100 μl of monoclonal antibody F7–26(1:10) for 15 minutes. After antibody is fixed on slide, these were applied 100 μl peroxidase-conjugated antimouse IgM (1: 200), incubated for 15 minutes and rinsed with PBS and applied DAB chromogen with hematoxylin-counterstaining. These slides were mounted slides for microscopy of MAB 3299 as apoptosis death marker that was independent of internucleosomal DNA fragmentation [13].

### Sodium MR imaging and histological comparison

Edge detection co-registration method was used to delineate boundaries and locate center point of tumor on MRI images and histologic sections with accuracy of 0.5 mm pixel resolution [14]. Semi-automated segmentation was used to distinguish location of different IC-Na signal intensities and corresponding tumor features [14]. Different cytomorphology features as histology end points were compared with the total sodium by SQ MRI and [Na]_i _by IR MRI signal intensities as following : 1) Ratio of intracellular and extracellular space by trichrome stain; 2) Tumor viability (intracellular space) using proliferation and apoptosis index expressed as number of proliferating and/or apoptotic cells per total number of cancer cells; 3) Cell cycle analysis based on DNA content (Feulgen stain); and active necrosis.

### Correlation of histological features with intracellular sodium MRI image signal intensity

The correlation was analyzed to co-register digitized histology maps with matched sodium MR images at different regions in the tissue to test null hypothesis. The hypothesis was that altered sodium signal intensities do correlate with immunohistology and cytopathologic biomarkers indicating different tumor features. Step down multivariate statistical analysis was performed to determine the order of immunohistolgical and pathologic parameters that correlate with the intracellular sodium (IC-Na) MRI signal [15].

## Results

### Animals

Out of total 30 animals, only 9 MNU induced animals (out of 15 animals) showed total 16 breast cancer tumors. Six animals (out of 15 animals) treated with Taxotere only and three animals served as untreated control group. These control untreated animals did not show any tumor. Other animals served as 3 sham and 3 water placebo treated second control animals. These six pre- and post-treated tumors were analyzed for IR intracellular sodium MRI and SQ total sodium MRI imaging. MRI data were correlated with histopathologic features and immunostaining parameters. Tumors were grouped for quantitative observations as control, obtained from MNU induced animals and compared with 16 tumor tissues obtained from Taxotere treated MNU induced animals.

### Validation of intracellular and extracellular sodium MRI signal intensity

A series of sodium agarose phantoms, showed distinct sodium MRI signal intensities at varying sodium concentrations as shown in Figure 2 top panel on left. T2 values were measured between the phantom and the extracellular fluid as shown in Table 1 and Figure 2. Figure 2 (panel at bottom on left) represents a plots of visibility as M_o_^SQ ^vs. sodium concentration for all concentrations of agarose used. The linear relationship between visibility as M_o_^SQ ^and M_o_^IR ^using relaxation constants and sodium concentration was observed with change in agarose concentration. The visibility of M_o_^IR ^increases more with % agarose concentration. Sodium MRI signal intensity showed linear relationship with sodium concentration as shown in Figure 2 on right panels.

**Table 1:** A comparison of slow and fast transverse relaxation times for different agarose concentrations and dependence of visibility Visibility^SQ ^(ΔM_o_/Δ[Na]) and visibility^IR ^(ΔMo/Δ [Na]) on agarose concentration. Visibility^SQ ^and visibility^IR ^for all concentrations of agarose were obtained using SQ and IR techniques. All transverse relaxation time constants *T*_*2s*_^*SE*^, *T*_*2s*_^*SE *^and *T*_*2s*_^*IR*^, *T*_*2s*_^*IR *^were determined by single quantum Hahn spin echo and IR pulse sequence on NMR.

% w/v Agarose	T_2s_^SE^	T_2s_^SE^	MRI Visibility^SQ^	T_2s_^IR^	T_2s_^IR^	MRI Visibility^IR^
10	12.4 ± 1.2	8.1 ± 0.48	1.75	13.7 ± 1.2	7.4 ± 0.5	3.45
20	7.5 ± 0.35	4.1 ± 0.31	1.65	8.4 ± 0.3	3.4 ± 0.3	4.50
30	7.1 ± 1.61	1.8 ± 0.04	1.5	6.4 ± 0.2	1.8 ± 0.04	4.70
40	3.4 ± 0.1	1.2 ± 0.04	1.4	4.6 ± 0.2	1.2 ± 0.1	7.22

### Sodium Inversion Recovery nulls signal from selected T1 range

**Figure 3:**
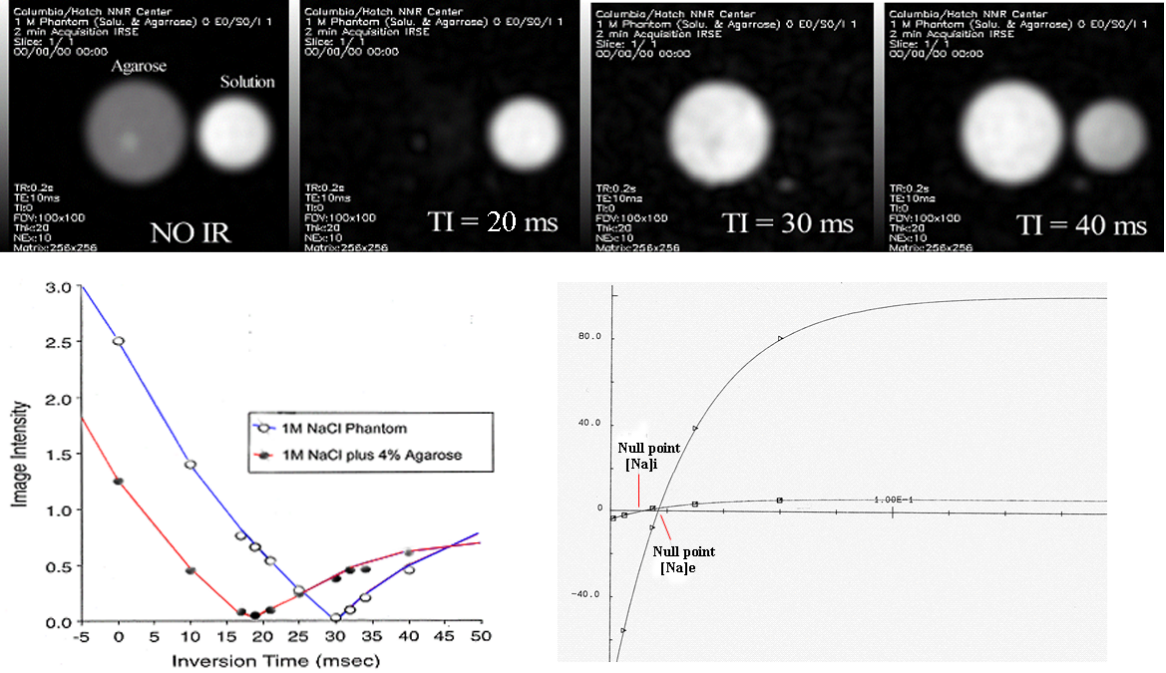
(Top row) The phantom consists of two tubes, one is 1 M NaCl in solution and the other is 1 M NaCl in 4% agarose (Panel A). They simulate extracellular and intracellular sodium, respectively without use of inversion recovery pulse. It can be seen that an inversion time of TI = 20 ms completely suppressed intracellular sodium (Panel B) and TI = 30 ms completely suppressed the extracellular sodium signal from the tube containing NaCl in solution (Panel C). At TI = 40 ms partial suppression of extracellular sodium is highlighted (Panel D). (Middle row) Two 1 M NaCl phantom image intensities (with 4% agarose, open circles; and, without 4% agarose, closed circle) were plotted during single quantum acquisition and for 9 different inversion recovery times (TI) showed two distinct null points shown at right.

The inversion recovery (IR) pulse sequence suppressed the signal from Na nuclei with long T1 relaxation constants (free EC Na). For it, the inversion time (the time between the 180° and 90° pulses in the IR pulse sequence) was calculated as (ln 2)(T_1_^ex^), where T_1_^ex ^was composite longitudinal relaxation time. It generated an intracellular (IC) weighted sodium signal and MRI image. The inversion time selectively suppressed the signal from either long T1 (EC-Na) or short T1 (bound IC-Na) in agarose phantoms at lesser or more than the optimal TI choice as shown in Figure 3. At inversion time TI = 30 msec, the long T1 signal from free EC-Na in NaCl phantom was suppressed completely. Two 1 M NaCl phantoms (with 4% agarose shown as ○ open circles; and, without 4% agarose shown as ● closed circles) were examined during both single quantum MRI acquisition and inversion recovery MRI acquisition for nine different inversion recovery times. Data points represented image intensities while the lines showed theoretical relationships and two null points assuming TI = 27.4 and 43.3 msec for sodium images [5].

### Application of Sodium Inversion Recovery method to breast Tumors

**Figure 4:**
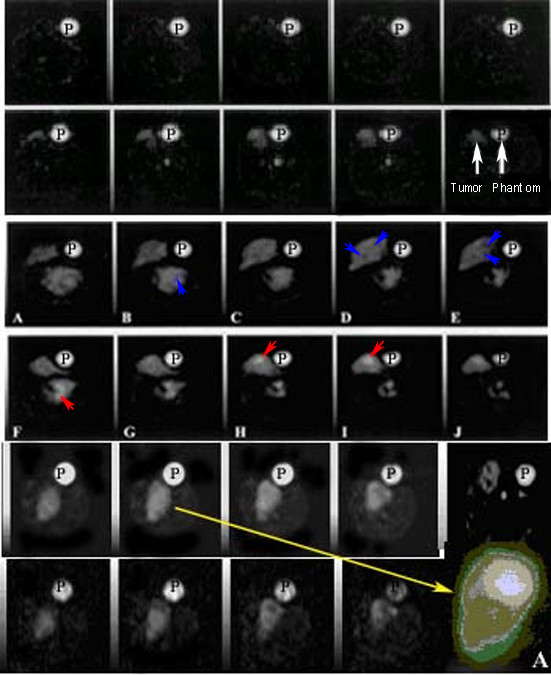
Taxotere chemosensitivity effect is compared on SQ images of pre- and post Taxotere treatment (first vs second rows on top). Panel on second row on rightmost image, represents the tumor SQ image (left arrow) and phantom (right arrow). Third and fourth rows represent the panels showing contrast (arrows) on contiguous intracellular sodium MRI images as "IR" images of pre- and post Taxotere treatment respectively by inversion recovery method. Panels in fifth and sixth rows represent the intracellular sodium signal intensity increases (arrows) in some areas representing active proliferation or resting viable cells. Loss of signal in late apoptosis rich regions and increased tumor areas can be seen as hypointense areas (panels in fifth row vs sixth row for pre- and post-injection of taxotere). An enlarged "IR" image (panel in sixth row) of tumor (on rightmost insert A) after segmentation image processing is shown with different bright, gray and darker pixel signal intensities.

Single quantum (SQ) sodium images (Figure 4, panels in rows 1 and 2) showed distinct bright tumor on the SQ images. IR pulse sequence perhaps suppressed the extracellular sodium at optimal inversion time (TI) applied on rat model of breast cancer. Optimum inversion time (TI_opt_) was chosen 25–30 msec based on quantitative difference in spin-lattice relaxation constants (T1). At this inversion time, the signal was totally nulled for cyst regions e.g. a tumor sac that was predominantly fluid, showed up darker shown as blue arrows in Figure 4, right panels D, E in rows 3 and 4. It enhanced the visibility of semi-solid or proliferative tumor regions that appeared relatively brighter in comparison to the rest of rat body shown as red arrows in Figure 4, panels F, H, I. It may be presumably due to the higher intracellular (IC) sodium in proliferative regions in tumor vs normal breast tissue. IR sodium images (Figure 4, image panels in rows 3 and 4) showed distinct tumor heterogeneity i.e. cyst, solid tumors but poor tumor boundaries.

Breast tumors overall showed around 2 – 3 times higher intracellular(IC) sodium MRI signal over normal body IC sodium MRI signal intensity in baseline control animals shown in Table 2 and Figure 4 (panels in rows 5 and 6).

**Table 2:** Comparison of tumor characteristics in different regions is shown in pre-drug treated tumor^@ ^and post-treated tumor^# ^by sodium MRI image intensity and histology, histo-immunology parameters as shown in Figures 5 and 6. By using eyepiece-micrometer square counter (100 squares per high power field), necrosis*(<25% cells in micrometer square), viable cells**(<60% cells in micrometer square) and apoptosis*** (20–40 apoptotic nuclei in HPF) and cyst space****(<100 μm) per HPF were premalignant histology characteristics. IC/EC space (% space in HPF), necrosis, viable cells are shown as number of micrometer squares with <25% necrosis area in HPF by histology. Apoptotic index (A.I.) and proliferation index (P.I.) are shown as average number of apoptotic nuclei per HPF and number of mitotic figures per HPF. S-DNA histogram area was measured by CAS 200 system in arbitrary units. Single strand-DNA mAb area was measured in digital images by Optimas 6.5 and ss-DNA mAb density was measured in arbitrary units of photomultiplier scanner.

Pre-drug treated^@ ^Post-drug treated^#^	Sodium MRI signal intensityd		Histology (in HPF)	A.I. (KI-67)	P.I. (in HPF)	S-DNA (histogram)	ss-DNA mAb (density units)
						
Tumor feature	IR	SQ	(CAS)				
Control (n = 41)	4.25 ± 1.0	5.27 ± 2.0	-	-	-	-	-
							
Sham (n = 40)	4.30 ± 0.2	5.38 ± 1.0					
							
Tumor area(mm^2^;n = 16)^@^	4.56 ± 0.30	4.67 ± 0.30	4.49 ± 0.2	-	-	3.6 ± 0.2	4.2 ± 0.2
Tumor area(mm^2^;n = 3)^#^	4.60 ± 0.29	4.61 ± 0.26	4.42 ± 0.3	-	-	3.5 ± 0.3	4.3 ± 0.3
							
IC/EC space^@^	60–70		65–76	-	-	-	-
IC/EC space^#^	80–95		60–70	-	-	-	-
							
Necrosis*(squares)^@^	gray		56 ± 32	-	270	M-DNA	-
Necrosis*(squares)^#^	bright		50 ± 23	-	120	M-DNA	-
							
Viable**(squares)^@^	dark		65 ± 21	-	-	-	-
Viable**(squares)^#^	dark		76 ± 11	-	-	-	-
							
Apoptosis***(nuclei)^@^	bright		40 ± 12	150	-	S-DNA	159 ± 12
Apoptosis***(nuclei)^#^	bright		50 ± 13	160	-	M or S-DNA	129 ± 20
							
Cyst****(size in μm)^@^	gray		120 ± 29	-	-	-	-
Cyst****(size in μm)^#^	gray		80 ± 22	-	-	-	-

### IR-Na signal intensity changes and immunostaining features in post Taxotere treated tumors

Comparison between pre- and post-treatment single quantum (SQ) sodium images (Figure 4, panels in rows 1 vs 2) showed increased size as tumor boundaries but poor tumor heterogeneity. However, the tumor showed up as bright on the post-treatment SQ image. Viable, active proliferative necrosis and apoptosis regions showed up hyperintense on post-treated IR images but unchanged or hypointense on post-treated SQ images. Cysts, extracellular space showed hypointense regions on post-treatment IR images but hyperintense on IR images as shown with arrows in Figure 4 (rows 5 and 6). These enhanced IR signal intensities after Taxotere 24 hour post-treatment were associated with both enhanced apoptotic index (9%) and reduced proliferation index (8.6 vs 2.2; 4 fold decrease over pre-treated tumors) in these regions based on NIH Image J pixel reader counts at different colored locations in post-segmented tumor as shown on Figure 4 (rightmost insert A in row 6). Using calibration graph, the high sodium signal intensities in active regions were measured relative to phantom (P) as shown in Figure 4 and Table 3 showing distribution of sodium in different tumor regions.

**Table 3:** Different SQ and IR sodium MRI signal intensities and sodium concentrations (mean ± SD) are shown by using NIH Image J pixel density reader at different x, y pixel positions in tumor different regions of active necrosis, viable, apoptosis, cyst regions shown in a representative tumor in Figure 6. Untreated 3 control, 3 sham animals, and 3 water placebo injected animals; counterpart pre-treated 3 tumor bearing animals on day 1 and post-treated 6 tumor bearing animals on day 2 after taxotere injection showed distinct SQ and IR MRI sodium signal intensities in excised tumors (3 pretreated tumors and 16 post-treated tumors) at their different n = 160 tumor sites in pre-treated and n = 160 post-treated tumor sites showed comparable changes in SQ and IR sodium signal intensities. For validation, sodium MRI signal intensities changes were compared with histology and immunostaining methods in different tumor x, y positions (n = 160) showing different features in 24 hours post-treated animal tumors. % difference of sodium signal intensity represents comparison between control vs. sham or water placebo; pre-treated vs post-treated tumor sites. *Apoptosis represents single large nuclear bead inside the cytoplasm during terminal stage without nuclear fragmentation. Mitotic figures represent number of visible mitotic spindles observed per high field under microscope.

Tumors (n = 16) features	SQ Sodium MRI		IR Sodium MRI		% difference		
				
	signal intensity A.U.	concentration (mM)	signal intensity A.U.	concentration (mM)	SQ	IR	Mitotic figures (per HPF)
Contol Phantom:	9.5 ± 0.3	100	4.2 ± 0.5	10			
**Day 1(0 hour):**							
Control(n = 41)sites	5.6 ± 0.2	58.9 ± 2.1	4.5 ± 0.1	10.7 ± 1.0			
Sham(n = 40)sites	5.5 ± 0.6	57.8 ± 6.3	4.6 ± 0.2	10.9 ± 2.1	1.7 ± 0.5		
Water placebo(n = 40)	5.9 ± 0.8	62.0 ± 8.4	3.3 ± 0.3	7.8 ± 0.7	5.3 ± 2		
Pre-treatment							
Tumor regions (n = 160 sites)							2.4 ± 0.6
Viable	6.8 ± 1.1	71.5 ± 11.6	6.1 ± 0.8	14.5 ± 1.9			
Active necrosis	7.6 ± 1.4	79.9 ± 9.4	8.6 ± 1.0	20.5 ± 2.4			
Apoptosis	6.2 ± 0.9	65.2 ± 9.2	10.8 ± 1.2	26 ± 3			
Cyst	19.5 ± 2.3	205.1 ± 24.2	7.2 ± 0.7	17.1 ± 1.6			
*Apoptosis	6.2 ± 1.6	65.2 ± 16.8	10.3 ± 1.4	24.5 ± 3.3			
**Day 2(24 hours):**							
Control(n = 41)sites	4.7 ± 0.2	49.4 ± 2.1	3.5 ± 0.4	8.3 ± 0.9	16.0 ± 0.2	22 ± 0.6	
Sham(n = 40)sites	6.8 ± 0.8	71.5 ± 8.4	4.2 ± 1.1	10 ± 2.6	23.6 ± 3.3	8.6 ± 0.8	
Taxotere injected (n = 40 sites)	7.9 ± 0.4	83.1 ± 4.2	5.0 ± 0.4	11.9 ± 0.9	33.8 ± 0.5	51.5 ± 3.3	
24 hr Post treated							
Tumor regions (n = 160 sites)							8.5 ± 0.5
Viable	6.2 ± 1.6	65.2 ± 16.8	7.6 ± 1.2	18.0 ± 2.8	8.8 ± 0.5	24.5 ± 0.4	
Active necrosis	6.8 ± 1.2	71.5 ± 12.6	9.1 ± 1.0	21.6 ± 2.4	10.5 ± 1.4	5.8 ± 0.5	
Apoptosis	6.4 ± 0.9	67.3 ± 9.2	18.8 ± 1.7	44.7 ± 4.0	3.2 ± 0.0	74.0 ± 4.1	
Cyst	22.6 ± 2.6	237.7 ± 27.3	6.2 ± 1	14.7 ± 4.3	15.9 ± 1.3	13.8 ± 0.58	

**Figure 5:**
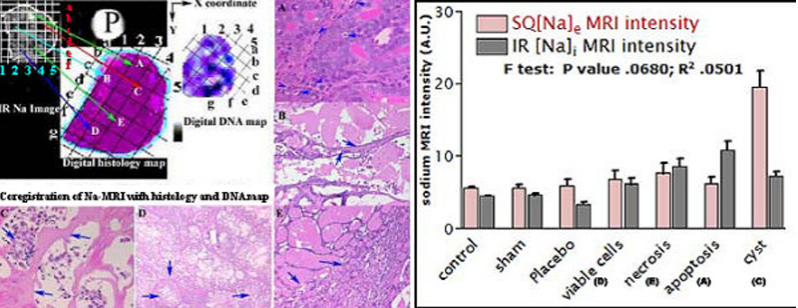
The figure shows the stereotactic co-registration method of IR sodium tumor image, its counterpart histology and DNA ploidy map at different x- and y- co-ordinate locations shown as 1–5 and a-g for different areas. Notice the matched tumor delineated areas on three digitized images and corresponding features shown with arrows for high % apoptosis (A), high % EC volume (B), cyst (C), % viable cells and proliferation (D), % necrosis (E) in high power fields. The tumor IR sodium MR image appears hypointense for cyst and high EC regions and hyperintense for active proliferation while isointense for necrosis. On right panel, sodium signal intensities are shown for 100 mM SQ sodium phantom and 10 mM IR sodium phantom along with relative MRI signal intensities in the different tumor regions.

Co-registration indicated the possibility of edge detection and point-by-point (equal square size) stereotactic matched quantitation of sodium MRI images with histology and immunostaining maps as shown by squares 1–5 on × axis and squares a-g on y axis in Figure 5. The sodium MRI signal intensities were measured relative with phantom (P) at different tumor locations under high power field shown as apoptosis (A), high EC volume (B), cyst (C), proliferation and viable cells (D) and necrosis (E) in Figure 5 and Table 3. However, DNA cycle maps indicated different features but not conclusive while sodium MRI signal intensities differed by SQ and IR methods mainly for apoptosis (A) and cyst (C) along with other tumor features as shown in Figure 5 (rightmost panel) and Table 3.

**Figure 6:**
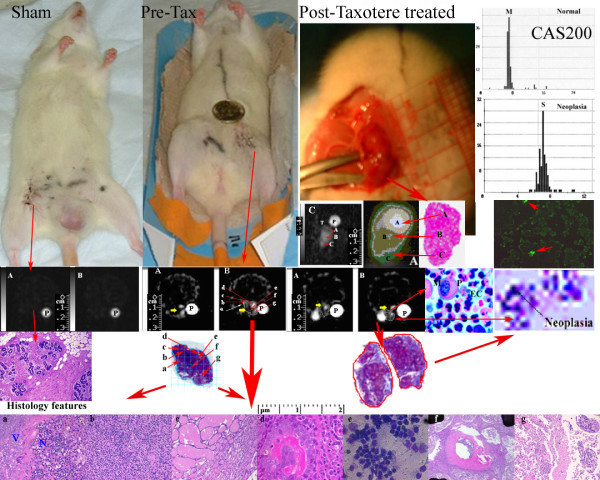
The sham control, pre-treatment and Taxotere post-treated animals (top panels on left) show tumors as SQ sodium (A) and IR sodium (B) images at 0 hr pre-Taxotere and 24 hours post-Taxotere treatment (panels A and B on second row). On third row on left, control tumor histology shows normal vesicles. Pre- and post treated excised tumor histology by trichrome staining is shown with delineated area. On fourth row on left, the excised tumor histology features in high power fields are shown with arrows (active viable cells (a), proliferation (b), necrosis (c), apoptosis (d), mitosis (e), fibrous cyst (f), and infiltrating ductile carcinonoma (g) in different x- and y- coordinate locations after coregistration with IR sodium images. On right, panels on top show a IR sodium MR image before (C) and after non-parametric segmentation by Optimas 6.5 to highlight the different signal intensities that appeared hyperintense, isointense, and gray-green colored on segmented image and histology features showed them as apoptosis (A), necrosis (B) and neoplasia (C). On right, second row shows corresponding S DNA histograms of neoplasia features by CAS 200 (panels on top), apoptosis staining (panel with green stain). On right, third row shows a post-Taxotere treated tumor histology by pentachrome stain to highlight mitotic figures (M) with active PMN cells (P) and high EC volume (EC) and corresponding digitized map of DNA cycle, with neoplasia shown as arrow.

MRI images showed isointense IC sodium signal intensities in 3 sham rats following placebo water injections for comparison with 3 pre-treatment MNU breast tumors. In control animals, histology and sodium MRI images were normal as shown in left panels of two rows in Figure 6. MNU induced breast tumor sites showed increased IC sodium signal intensity (2–5 fold increase, p < 0.01; n = 16) compared with matched water placebo-sham control sites shown in Figure 6 (left panels in second row). In 3/9 tumor bearing pre-treated animals (second column) showed main features as following: 1. Increased IR-Na signal intensities (yellow arrows) with corresponding immunostained areas of late S-phase in their excised tumors; 2. Tetraploid nuclei or actively dividing nuclei in G2 phase with M and S histogram indicating the presence of more necrotic cells with late S phase completion in viable cells; 3. Neoplasia stage appeared as gray with isointense IR-Na signal during their late S-phase shown as distinct S-DNA histogram in Figure 6 (rightmost panel in second row) with corresponding S-DNA cycle map in Figure 6 (rightmost panel in fourth row); 4. Distinct excised tumor histology features in panels under high power fields as active viable cells (a), proliferation (b), necrosis (c), apoptosis (d), mitosis (e), fibrous cyst (f), and infiltrating ductile carcinonoma (g) in different x- and y- coordinate locations after point-by-point coregistration with IR sodium image (arrows a-g). In 24 hours Taxotere treated animals showed main features as following: 1. Increased IR-Na signal intensities (yellow arrows) were associated with tumor cells arrested in G0/G1 or early S phase (diploid or anoploid cells), neoplasia stage with S-DNA histogram patterns as shown in Table 2 and Figure 6 (rightmost panel in fourth row); 2. Hyperintense, isointense, and gray-green features on color-coded segmented IC-Na image (C panel in second row) with tumor histology features as apoptosis (A), necrosis (B) and neoplasia (C); 3. Reduced gray tumor area on sodium MRI image perhaps due to more bright apoptosis rich areas (yellow arrows on panels A and B) with corresponding histology features (delineated tumor area); 4. Longer early S-phase of resting tumor cells; 5. Reduced diploidy or aneuploidy in dividing tumor cells undergoing cell division (in 80% tumors); 6. Decreased tumor viability as mean proliferation index (PI); and 7. Reduced proliferation index.

**Figure 7:**
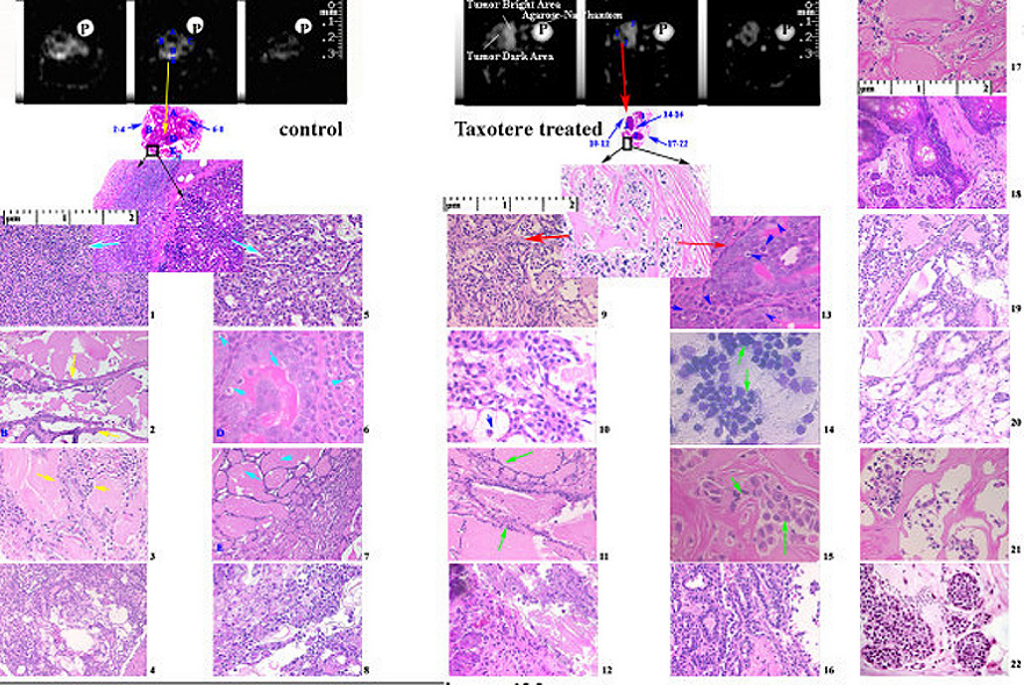
The figure represents a comparison of different tumor pre-malignant and malignant features in coregistered sodium IR images (panels in first row with phantom P) and their counterpart histology in low power (second row). Notice the heterogeneous regions of tumor and corresponding distinct histopathology. At some selected locations, relative IR sodium MR signal intensities* (A.U.) with reference to sodium phantom P (50 A.U. in top panels) are shown as numbers and histograms (see Figure 8) as distinct values for different tumor stages with different tumor features by NIH Image J 1.63. Panels (1–22) show high power optical microscopy (images 1–8) in control 0 hour (on left) and post-Taxotere 24 hours treated tumors (images 9–22 on right) with distinct features shown by arrows.

In excised pre-treated tumor histology sections, number of cells with distinct histology features per HPF (in mm^2^) were: mitotic figures (MI) (2.4 ± 0.6 in mm^2^), necrosis (56 ± 32 in mm^2^), viable cells (65 ± 21 in mm^2^) with early phase of apoptosis (fragmented nuclei with smooth call membrane) (40 ± 12 in mm^2^) in 100 small squares per high power field (HPF), at magnification × 400 including pre-malignant stages of ductal hyperplacia, intraductal proliferation in each specimen as shown in Figure 7 with Tables 2 and 4. After Taxotere treatment, excised tumor cells showed the number of cells with histology features per HPF (in mm^2^) were: mitotic figures (MI) (8.5 ± 0.5 in mm^2^), necrosis (50 ± 23 in mm^2^), viable cells (76 ± 11 in mm^2^) and apoptosis (50 ± 13 in mm^2^) in 100 small squares per high power field (HPF), at magnification × 400 showing pre-malignant stages, hyperplacia, proliferation cells with PI decrease 4-fold from 270 per mm^2 ^(per HPF) to 120 per mm^2 ^(per HPF) as shown in Tables 2 and 4. However, the biological significance could not be assessed with these observations. Different tumor malignant regions showed similar features in pre-treatment and post-treatment tumors rich with papillary or invasive comado and cribriform carcinoma, sarcoma or neoplacia and late apoptosis (tiny beaded nuclei with distorted cell membrane) and lower mean PI in Taxotere treated breast tumors as shown in Figure 7 and Table 4.

**Table 4:** Histological characteristics of MNU induced rat breast pre-treatment tumors under magnification × 400 microscopy equipped with micrometer. Different premalignancy and malignant characteristics and cytomorphology characteristics were co-registered with IR sodium signal intensities. Level of brightness is shown as + sign on MRI gray scale.

Sodium MRI signal Intensity		Histomorphology	Tumor characteristics
SQ Na MRI	IR Na MRI	Tumor features	Tumor stage

			Pre-malignant stage:
isointense dark/gray	++ +	Viable Active necrosis	Intraductal proliferation, ductal hyperplacia, apoptosis
			Malignant stage:
gray	++++	apoptosis	Transformation stage
+++	bright/gray	apoptosis	Carcinoma (papillary; invasive comedo and cribriform)
++++	dark	Cyst	sarcoma, neoplasia

Main findings were: 1. The number of apoptotic cells per mm^2 ^(HPF) i.e. apoptotic index (AI) was increased 10%; ~150 per mm^2 ^(HPF) vs ~-160 per mm^2 ^(HPF) in pre- and post-Taxotere treated tumors associated with enhanced intracellular IR sodium signal intensity by MRI in highly viable cell areas rich with pre-malignant stages; 2. Active single cell necrosis regions as isointense IC-Na signal intensity showing chemotherapeutic effect while advanced necrosis showed gray IC-Na signal intensity (in 6/6 animals; on 9/54 tissue slices). This was suggestive of increased IC sodium in 6 post-treatment animals as shown in Figures 6 and 7.

The tumors exhibited decreased IC sodium intensity (22 ± 4%) following chemotherapy (p < 0.001; n = 40), a response significantly different (p < 0.002) than that in control as shown in Figure 6. The reduced IC sodium intensity change (-5 ± 4%; n = 41 sites) in control animals vs. placebo-water injection perhaps was due to its water-insensitive characteristic (as shown in Figure 3 on panel B and Figure 6) but significant in control vs. placebo water injected animals (28 ± 2%; n = 40 sites) after 24 hrs perhaps due to inflammatory processes at sham sites. The average IC sodium signal intensity at Taxotere injected plain tissue sites showed elevated IC-Na signal compared to water placebo-sham control sites (30 ± 4%, p < 0.05; n = 40 sites) as shown in Table 3. Taxotere treatment showed a 34 ± 3% IC sodium signal intensity increase over pre-Taxotere treated tumor (p < 0.001; n = 40; Figure 4 panels right vs left on fifth vs sixth row).

The sham control, pre-treated and Taxotere post-treated animals bearing MNU induced tumor are shown on top row in Figure 6. Their SQ and IR sodium images and histology with immunostaining features showed characteristic signal intensities as shown in Table 3. SQ sodium images displayed better tumor area (panels in 1 and 2 rows) while IR sodium images displayed tumor heterogeneous features as shown in Figure 4 (panels in 3 to 6 rows) and Figure 7 (panels on top row).

### IR-Na signal intensity changes and extracellular vs. intracellular space

Histology and the pentachrome staining measured the ratio of intracellular vs. extracellular space (EC) as shown (red arrow) in the panel on third row with polymorph cells (P) rich with mitotic figures (M) in Figure 6. Pentachrome staining further demonstrated the distribution of extracellular space (EC) shown as lighter and intracellular space (IC) as darker in 54/58 (in 90%) histology slides. The % IC/EC space ratio indicated viable cells and cells at risk or dying cells. Viable cells showed the average ratio '% IC/EC' ratio as 60–80%. It represented the number of viable cells and higher mitotic Index. The dying cells showed higher '% EC/IC ratio' 70% or above with higher Apoptotic Index (AI) of cells possibly in late apoptosis phase. The extracellular space showed cell debris, dying cells with fluids. It contributed minimum to intracellular space. Gross morphology further determined the intracellular contents including collagen, extracellular matrix, cyst fluid (color ranging lighter for cyst to darker for collagen) as shown with arrows in IR sodium MRI image. The comparison of IC/EC ratio with the MRI images is shown in Table 2. Areas comprising less than 30% IC space showed up as dark to hypointense regions whereas areas comprising 30–50% intracellular space showed up hypointense to isointense on IC-Na MRI images. More than 50% IC space (i.e. less than 50% EC) showed up brightest or hyperintense on IR-Na MRI images. Intracellular sodium images (IR-Na) showed medium to bright MRI areas in (36/54 tumor image slices) representing more than 50% IC space. Low intracellular sodium signal intensity in MRI images represented less than 40% IC space in the tumor regions. Control tumors showed the more viable cells and more intracellular space than the post-Taxotere treated tumors by H&E staining.

The apoptotic index was increased marginally in post-treated breast tumors (160 per mm^2^) vs. control tumors (150 per mm^2^) that indicated the negative correlation between intracellular sodium signal intensity by MRI and cell viability as shown in Tables 2 and 4. Single cell necrosis showed enhanced IC-Na sodium signal intensity depending on the degree of necrosis while extensive late necrosis showed low SQ Na MRI signal intensity as shown in Table 4 and Figure 6, perhaps due to chemosensitivity effect, ischemia or increased tumor vascular supply. Large areas of late necrosis (low signal intensity) were not observed within 24 hours after initiation of Taxotere chemotherapy in breast cancer.

### Histology and Immunostaining features

A "touch slide preparation" of 4 micron thick serial sections by 10% Fuelgen staining (41/48 tumor sites) determined the s-DNA content (in 3 control tumor tissues and 16 Texotere treated tumor tissues) as shown in rightmost panel on third row in Figure 6. The 4 micron thick histology section staining by H&E showed histology features of necrosis, apoptosis, proliferation, mitotic rate (number of mitotic figures per mm^2^) and amount of apoptosis (number of apoptotic nuclei per mm^2^) in 36/48 (75%) tumor sites.

### Chemosensitivity assay of Taxotere effect using cell proliferation

Ten explanted Taxotere-treated tumors showed enhanced IR-Na MRI signal intensities by microimaging of tumors at 0 hour and after 24 hours. Central necrosis appeared dark in center and bright peripherypresentation on the IR image as shown in second row on Figure 6. These tumors were uniformly bright on the IR image. Based on histology, these post-treatment tumors showed neoplastic features rich with cyst or absence of cell nuclei, apoptotic cells and proliferation rich necrosis zones as shown in bottom row in Figure 6. An outermost peripheral zone appeared viable tumor tissue. Apoptosis cells were distributed in unspecific manner but apoptosis rich zones were common between viable (V) zones and necrosis (N) rich zones seen in HPF shown in Figure 6 bottom row (see panel a) and Figure 7 (see panels 13).

Under HPFs, the untreated tumors showed high number of mitotic figures (see Figure 6, panel b and Figure 7 panel 1). The mitotic figures measured up to >20 per HPF in the viable zones of tumor. Mitotic figures were altered significantly (p < 0.0001) in the Taxotere-treated 9 tumors (8.5 ± 0.5 per field; n = 160 HPFs) as shown in Figure 7 (see panel 9) and Table 2. All post-treatment tumors showed a significant inverse correlation (p < 0.02) between the reduced mitotic figures and the relative increase in IR sodium signal intensity on IR images.

### Correlation of Image Configuration with Histology and Immunostaining

Pre-Taxotere and post-Taxotere treated tumors showed enhanced SQ- and IR-MRI signal intensities (panels A and B) over the untreated matched tissue (panel A) and sham-control tissue (panel B) on their sodium MRI images as shown in Figure 6. In tumors with small or absent non-viable centers, the IR-MRI images showed uniformly bright regions. The non-viable centers were comparatively smaller than necrosis rich regions. The chemosensitivity effect of Taxotere (enhanced sodium MR image intensity) was evident at histology matched locations of apoptosis (A), neoplastic (B) tumor features rich with cyst or absence of cell nuclei (C), and proliferation rich (D) and necrosis (E) zones as shown in Figures 5 and 6. Taxotere 24 hour post-treatment showed the increased bright area on the IR MRI image as shown in Figure 6 (panels A and B on second row). The IR MRI image in panel C showed different tumor features as dark center indicating late necrosis region (A), hyperintense region indicating apoptosis (B) and lower hypointense region indicating viable cells (C) as shown with red arrows in corresponding post-segmented IR MRI image (A) and its histology matched section. The 24 hours post Taxotere treated tumors showed unchanged IR MRI signal intensities at histology matched necrosis but increased IR MRI signal intensities at histology matched apoptosis showed as yellow arrows on panels A and B (see pre Taxotere and post taxotere images) following the Taxotere drug administration as shown in Figure 6. However, neoplasia and apoptosis were identified by CAS 200 and fluorescent ss-DNA monoclonal antibody staining showed apoptosis cells (next to the viable region) as brightest and the cells closer to the smaller nonviable center were less bright as shown in Figure 6 (red arrows on rightmost panel in third row). The delineation reflected measurement accuracy on histology and matched IR MRI image is shown with red arrows in Figure 6.

### Microimaging and tumor characterization

**Figure 8:**
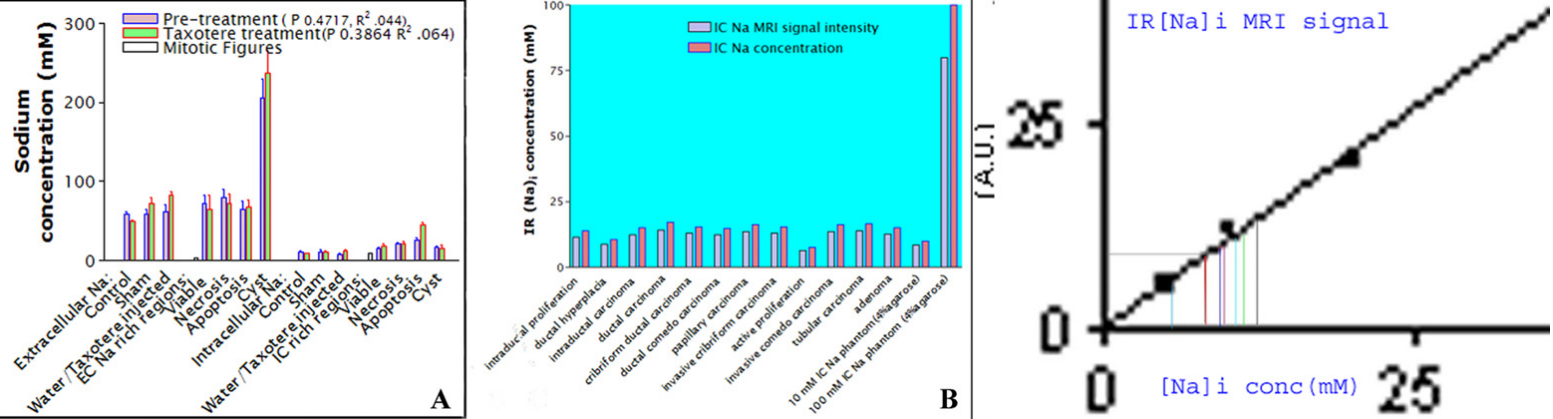
The sodium IR MRI signal intensities and calculated IR sodium concentrations are shown as histograms for pre-malignant tumor features shown in Figure 7. In left panel A, different intracellular sodium MRI signal intensities and concentrations indicated for [intraductal proliferation 11.5(2); ductal hyperplacia 8.8 (3); apoptosis with intraductal carcinoma 12.4* (6,13) and active proliferation 6.2* (2); ductal carcinoma 14.2* (17); cribriform ductal carcinoma 12.8* (7); ductal comedo carcinoma 12.2* (4)]; Malignant features [papillary carcinoma 13.5* (9,11); invasive cribriform carcinoma 12.8* (18); invasive comedo carcinoma 13.5* (22); tubular carcinoma 13.8*; adenoma 12.5* (2,8)]. Tumor different histology features under high power fields are shown in brackets correspond with histology regions in Figure 7. The panel B, in center represents the match of IC sodium concentrations with IC sodium MRI signal intensities. In different tumor regions, IC sodium MRI concentrations were calculated relative to the phantom MRI signal intensities shown as linear curve in Figure 2. In the right panel C, IC sodium concentrations and MRI signal intensities at different tumor premalignant or malignant locations represent the possibility of quick assessment of tumor characterization with possible staging.

Sodium MRI signal intensities showed heterogeneous regions of pre-treated and post-treated tumors corresponding with characteristic pre-malignant and malignant different stages by cytomorphology as shown in Table 4 and Figure 7. However, sodium distinct IR MRI signal intensities and relative IC sodium concentrations using standard sodium phantom data of different tumor stages were evident but not conclusive. Main evident cytomorphic features were apoptosis, necrosis and cysts with characteristic sodium MRI signal intensities. Typical tumor histology features in both pre- and post Taxotere treatment appeared as intraductal proliferation, intraductal carcinoma with cribriform or comedo patterns as shown in Figure 7 (see panels 1 – 8 for pre-treatment tumors and panels 9–22 for post-treatment tumors). Some tumors showed papillary carcinoma, adenoma and neoplasia as shown in Figure 7 and Table 4. IC sodium MRI signal intensities and IC sodium concentrations showed marginal insignificant differences for tumor premalignant and malignant locations confirmed by histology as shown in Table 5 and Figures 7 and 8. However, relationship of drug treatment and tumor histology pre-malignant features was evident but non-specific and limited to one time point.

**Table 5:** Different IC Na MRI signal intensities represent the matched locations of different tumor premalignant and malignant stages on tumor histology sites as shown in Figure 7 in different panels for: pre-malignant features [intraductal proliferation 11.5(2); ductal hyperplacia 8.8 (3); apoptosis with intraductal carcinoma 12.4* (6,13) and active proliferation 6.2* (2); ductal carcinoma 14.2* (17); cribriform ductal carcinoma 12.8* (7); ductal comedo carcinoma 12.2* (4)]; Malignant features [papillary carcinoma 13.5* (9,11); invasive cribriform carcinoma 12.8* (18); invasive comedo carcinoma 13.5*(22); tubular carcinoma 13.8*; adenoma 12.5* (2,8)]. The IC Na concentrations were measured by using IC Na MRI phantom standard graph as shown in Figure 2.

Tumor feature	IC Na MRI signal intensity	IC Na concentration (mM)
intraductal proliferation	11.50	13.8
ductal hyperplacia	8.80	10.6
intraductal carcinoma	12.40	15.0
ductal carcinoma	14.20	17.0
cribriform ductal carcinoma	12.80	15.4
ductal comedo carcinoma	12.20	14.6
papillary carcinoma	13.50	16.2
invasive cribriform carcinoma	12.80	15.4
active proliferation	6.20	7.4
invasive comedo carcinoma	13.50	16.2
tubular carcinoma	13.80	16.6
adenoma	12.50	15.0
10 mM IC Na phantom	8.5	10.0
100 mM IC Na phantom	80.0	100.0

### Co-registration and comparison of histological and IR-Na sodium images

**Figure 9:**
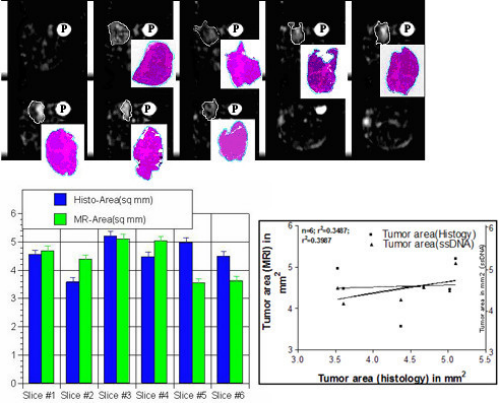
Comparison of delineated feature areas on intracellular sodium [Na]i images and histology digital images is shown at different levels for contiguous IR sodium MR slices (top row) and corresponding histology digital maps next to phantom (P). At bottom, each left bar represents histological area and each right bar represents delineated tumor area on both [Na]i image and histology digital images at different slice levels as shown in Table 5. Quantification of tumor area was done by Optimas 6.5 using sodium MRI and histology digital images. The tumor area was measured in mm^2 ^in one representative tumor shown in Figure 8. Tumor area was measured and correlated in contiguous 6 histology sections with matched IR images (r^2 ^= 0.3487) and scanned ss-DNA mAb digital images in mm^2 ^(r^2 ^= 0.3987). ss-DNA densities in arbitrary units were measured at different locations of the tumor digital images as shown in Figure 5.

In co-registered histological digital images and IR-Na sodium images showed % difference ~5% for tumor delineated area as shown in Table 6 and Figure 9 at the top row. Bar diagrams visualized the quick comparison of tumor areas. MRI imaging methods showed correlation with histology (r^2 ^= 0.34; n = 6) and s-DNA maps (r^2 ^= 0.39; n = 6) as shown in Figure 8 at bottom on right.

**Table 6:** Comparison of delineated feature areas on intracellular sodium [Na]i images and histology digital images is shown at different levels for contiguous IR sodium MR slices (top row) and corresponding histology digital maps next to phantom (P). At bottom, each left bar represents histological area and each right bar represents delineated tumor area on both [Na]i image and histology digital images at different slice levels as shown in Table 5. Quantification of tumor area was done by Optimas 6.5 using sodium MRI and histology digital images. The tumor area was measured in mm^2 ^in one representative tumor shown in Figure 8. Tumor area was measured and correlated in contiguous 6 histology sections with matched IR images (r^2 ^= 0.3487) and scanned ss-DNA mAb digital images in mm^2 ^(r^2 ^= 0.3987). ss-DNA densities in arbitrary units were measured at different locations of the tumor digital images as shown in Figure 5.

	Tumor Area Measurement_in images			
MRI slice	Sodium MRI	Histology	ss-DNA mAb	
	
	(in mm^2^)	(in mm^2^)	(in mm^2^)	(arbitrary units)
	(a)	(b)	(c)	
Slice 1	4.68	4.54	4.51	102.2
Slice 2	4.37	3.57	4.21	118.9
Slice 3	5.10	5.20	5.10	114.6
Slice 4	5.02	4.47	4.44	97.9
Slice 5	3.53	4.97	4.50	106.9
Slice 6	3.61	4.48	4.12	142.9

Statistical analysis suggested main determinants in the following order: tumor area by histology > tumor area s-DNA map > apoptosis index (immunostaining) > cell viability parameters associated with increased sodium MR signal intensity.

## Discussion

The study reports an application of a novel sodium MRI imaging method to stage breast cancer and monitoring Taxotere treatment response. This approach has significant potential as a tool for exploring breast cancer biologic features and as a clinical imaging approach. It describes sodium MRI inversion recovery pulse sequence and single quantum Hahn spin-echo method, their validation and application in tumor characterization in rapid sodium MRI *in vivo *monitoring of Taxotere chemotherapeutic response and breast tumor cytomorphology features.

Sodium concentration vs MRI MQ^IR ^signal intensity relationship was reliable in phantom experiments [8]. However, at sub-physiological levels, such relationship in tumor propogation is still not known. We believe that tumor cell destruction may cause intracellular sodium increase up to 2–4 times based on sodium extracellular space ~0.15 of total water space. Previous reports suggested selective suppression of extracellular sodium signal due to its high concentration by use of multiple-quantum, triple-quantum filter with little success to suppress selectively extracellular or intracellular sodium with or without use of contrast agent [13-18]. However, noninvasive sodium MRI methods without contrast agent for imaging cancer tumors and other tissues are emerging as new methods.

Present study focused on quantitative approach to demonstrate possible correlation of single quantum and *in vivo *sodium MRI inversion recovery (IR) image signal intensities with MNU induced breast cancer histology and immunostaining features to monitor Taxotere chemosensitivity. Sodium MRI and tumor cytomorphology features appeared relevant as previously reported MNU rat model closely simulated with human breast cancer [19-22].

The present study indicated the reliability and possibility of correlation between sodium heterogeneity and pre-malignancy with improved both spatial resolution and in-plane resolution up to some extent. Main problems were identified as the poor signal-to-noise ratio and molecular diffusion during the measurement or encoding. Sensitivity was enhanced by using small high temperature sensitive micro-coil receivers. The problem of molecular diffusion was minimized with strong magnetic field 4.23 T imager suitable for clinical imaging equipped gradients to encode spatial information quickly. Previously, different transverse relaxation constants and MR signal intensities of different sodium concentrations in model solutions indicated the possibility of sodium measurements in breast tumor at different pre-malignant or malignant locations [23,24].

The higher total sodium signal-to-noise ratio (SNR) of SQ pulse sequence in Na-MRI was an advantage over the IR pulse sequence. Intracellular Na weighting was accomplished by applying this inversion recovery pulse sequence to null the signal from sodium nuclei with long T1 in tumors [24]. Both phantoms and tumor MRI experiments suggested global suppression by use of non-selective 180° pulse and TI = 25–30 msec and it confirmed our previous report [5]. Earlier maximum suppression was reported at a TI = 50 msec [8]. In present study, short T1 intracellular Na nuclei were used as *in vivo *marker of antineoplastic and chemosensitive effects against apoptosis in MNU induced breast tumors in rats.

The inversion recovery technique minimized the contribution of fat. However, it suffers from the limitation of maximum inversion recovery after applying 180° pulse due to sodium diffusion in molecular domains with different T1 values during the recovery from inversion pulse. Choice of short inversion time is not advantageous as it reduces the differences of null points of two sodium components at short repetition time (TR).

Premalignant and malignant lesions in rats developed by injection of MNU at 50 days old animals have shown several advantages [4]. The present study supports earlier reports of elevated intracellular sodium [Na]i in both benign and malignant breast tumors, neoplastic, well-differentiated and poorly differentiated tumors than in their normal tissue counterparts [24-26]. Ionic alterations are important events in malignant transformation, apoptosis and necrosis, and progression through the cell cycle. Antineoplastic agents increase the intracellular sodium ions. Antineoplastic drugs change cell cycle distribution and often lead to apoptosis and altered intracellular sodium [Na]_i _[27-30].

Taxotere injected doses were at the higher end of the maximum tolerable dose (MTD) range (40 nmol/L) for rat above the clinical levels (10 nmol/L) for 24 hours[21,22]. Present study exhibited comparison with placebo effect and control. These *in vivo *breast tissue studies and *in vitro *literature reports both corroborate on increased intracellular sodium MR image intensity following chemotherapy at subphysiological levels [20,21]. However, intervention of hypoxia or ouabain poisoning further increases [Na]_i _and IC-Na longitudinal T1 constants [20,21].

The assessment of Taxotere chemosensitivity effect was based on assumption that different tumor cell characteristics such as cell cycle during apoptosis, subcellular intracellular or extracellular (EC or IC) space, necrosis, cell viability, compartmentalization all affect the sodium diffusion and Taxotere restores intracellular sodium reserve. Earlier reports on correlation of short T1 constants and sodium signal intensities of interstitial intracellular sodium with apoptosis support our view significant in comparing the histology and MR images in tumors [25,27,28]. Different tumor features in different tumor regions were visualized on the 3-D IR image by setting the pixel intensity threshold to a point about 50% below the tumor peak intensity (see Figure 3, rightmost insert in bottom row).

The present study signified tumor features with cystic fluid spaces or cellular debris appeared as dark on the IR weighted images. Control tumor showed low IR sodium signal and larger non-viable center. These tumors served as positive control pre-Taxotere tumor. Post-Taxotere treated excised tumors showed different pre-malignancy and malignancy stages with apoptosis, necrosis and cystic fluid. Tumors with bright centers on MRI images appeared rich in early apoptosis nuclei (fragmented nuclei but smooth cell membranes) and central region with non-viable tissue. These results were suggestive of neoplasia having brighter apoptosis-rich regions and/or early necrosis regions as shown in Figure 6. Moreover, ploidy CAS 200 analysis and H & E stained slices also supported these observations.

Single-strand DNA monoclonal based method enhanced the power of detection for different stages of apoptosis in tumor that was consistent with other reports [16,25,27,28]. Other available histo-immunostaining methods may speculate better such as caspase based methods for different apoptosis staging in tumors [31,32]. The pixel intensity histogram pattern showed distinct main peak of S-DNA predominantly neoplasia. CAS 200 method was efficient in distinguishing cell cycle phases as S, M peaks [12].

Stereo-micrometric divisions on co-registered digital histology and IR images further enhanced dimensional accuracy with sufficient resolution to analyze the cellular details at different locations in the tumors. The % difference <5% between sodium MRI and histology observations suggested the possibility of diagnostic accuracy and correlation of IR sodium signal intensity with histology features. Spatial resolution of the image (~1 mm within each slice) enhanced the sensitivity and sodium MR signal intensity on images.

The present study has limitations. The relationship of sodium signal intensities and sodium concentrations in phantoms appears linear but *in vivo *tissues indicate poor sensitivity and need further improvement. Other limitation was very close sodium calculated values of different tumor features. It raises doubt of accuracy in predicting tumor stages. Since the tumor was excised after the final sodium MR imaging session that was not always the point of peak IR-Na image enhancement and tumor immunostaining methods were limited in postmortem analysis. The Taxotere treatment was limited to 24 hours. Origin of the bright entities on the IR images seems to be affected by several subphysiological factors and partial volume averaging (PVA) as possible association. Comparison between pre- and post treated tumor images and histology pre-malignant and malignant features were not conclusive. It may suffer due to different animal tumors and semi-automated or operator bias in the applied methods. A more detailed quantitative analysis is required to speculate biologic significance of different in IC-Na MRI signals for different specific tumor cell populations and specific sodium ion responses with molecular events induced by the chemotherapy.

The interaction of chemotherapy, cellular apoptosis and sodium MRI reflects the possibility of Na-MRI as clinical imaging possibility. Other purpose of present study was to analyze the association of sodium MR intensities with apoptosis and tumor histology features. In near future, sodium images can be co-registered with proton high resolution MRI images using double tuned probes. It seems a further advantage of IR technique that it does not require potentially toxic shift reagents as other sodium Na MRI methods use shift reagents [2,3,17,33-36].

## Conclusion

The present study showed the intracellular sodium weighted inversion recovery MR pulse scheme for breast tumor sodium MR imaging and comparison with total sodium weighted single quantum MRI imaging and histo-immunostaining chemotherapeutic assessment. The intracellular sodium in breast tumor appears as associated with neoplasia, apoptosis and tumor histology features.

## References

[B1] Boada FE, Shen GX, Chang SY, Thulborn KR (1997). Spectrally weighted twisted projection imaging: reducing T2 signal attenuation effects in fast three-dimensional sodium imaging. Magn Reson Med.

[B2] Ouwerkerk R, Bleich KB, Gillen JS, Pomper MG, Bottomley PA (2003). Tissue sodium concentration in human brain tumors as measured with 23Na MR imaging. Radiology.

[B3] Clayton DB, Lenkinski RE (2003). MR imaging of sodium in the human brain with a fast three-dimensional gradient-recalled-echo sequence at 4 T. Acad Radiol.

[B4] Thompson HJ, Singh M, McGinley J (2000). Classification of Premalignant and malignant lesions developing in the rat mammary gland after injection of sexually immature rats with 1-Methyl 1 Nitrosourea. J Mammary Gland Biology and Neoplasia.

[B5] Kline R, Wu EX, Petrylak DP, Szabolcs M, Alderson PO, Weisfeldt ML, Cannon PJ, Katz J (2000). Rapid *in vivo *monitoring of chemotherapeutic response using weighted sodium magnetic resonance imaging. Clin Cancer Res.

[B6] Teh BS, Vlachaki MT, Aguilar LK, Miles B, Ayala G, Lattime EC, Gerson SL (2000). Case study of combined gene and radiation therapy as an approach in the treatment of cancer. Gene therapy of cancer.

[B7] Weissleder R, Mahmood U (2001). Molecular imaging. Radiology.

[B8] Song HK, Wright AC, Wolf RL, Worli FW (2002). Multislice double inversion recovery pulse sequence for efficient black blood MRI. Magn Reson Med.

[B9] Liska J, Galbavy S, Macejova D, Zlatos J, Brtko J (2000). Histopathology of mammary tumors in female rats treated with 1-methyl-1-nitrosourea. Endocr Regul.

[B10] Shilkaitis A, Green A, Steele V, Lubet R, Kelloff G, Christov K (2000). Neoplastic transformation of mammary epithelial cells in rats is associated with decreased apoptotic cell death. Carcinogenesis.

[B11] Kimi K, Kumamoto H, Ooya K, Motegi K (2001). Immunohistochemical analysis of cell-cycle- and apoptosis-related factors in lining epithelium of odontogenic keratocysts. J Oral Pathol Med.

[B12] Lee S, Tolmachoff T, Marchevsky AM, Marchevsky AM, Bartels PH (1994). DNA content analysis. Image Analysis: A Primer for Pathologists.

[B13] Kohler S, Preibisch C, Nittka A, Hasse A (2001). Fast three-dimentional sodium imaging of human brain. MAGMA.

[B14] Uemura K, Toyama H, Baba S, Kimura Y, Senda M, Uchiyama A (2000). Generation of fractal dimension images and its application to automatic edge detection in brain MRI. Comput Med Imaging Graph.

[B15] Lin SP, Song SK, Miller JP, Ackerman JJ, Neil JJ (2001). Direct, longitudinal comparison of (1)H and (23) Na MRI after transition focal cerebral ischemia. Stroke.

[B16] Frankfurt OS, Krishan A (2001). Identification of apoptotic cells by formamide-induced DNA denaturation in condensed chromatin. J Histochem Cytochem.

[B17] Duvvuri U, Leigh JS, Reddy R (1999). Detection of residual quadruple interaction in the human breast in vivo using sodium-23 multiple quantum spectroscopy. J Magn Reson Imaging.

[B18] Miller JR, Zhang K, Ma QY, Mun IK, Jung KJ, Katz J, Face DW, Kountz DJ (1996). Superconducting receiver coils for sodium magnetic resonance imaging. IEEE Trans Biomed Eng.

[B19] Lu J, Jiang C, Mitrenga T, Cutter G, Thompson HJ (1996). Pathogenic characterization of 1-methyl-1-nitrosourea-induced mammary carcinoma in the rat. Carcinogenesis.

[B20] Thordarson G, Lee AV, MacCarty M, Van Horn K, Chu O, Chou YC (2001). Growth and characterization of N-methyl-N-nitrosourea-induced tumors in intact and ovariectomized rats. Carcinogenesis.

[B21] McGinley JN, Knott KK, Thompson HJ (2002). Semi-automated method of quantifying vasculature of 1-methyl-1-nitrosourea-induced rat mammary carcinomas using immunohistochemical detection. J Histochem Cytochem.

[B22] Knott KK, McGinley JN, Lubet RA, Steele VE, Thompson HJ (2001). Effect of aromatase inhibitor vorozole on esterogen and progesterone receptor content of rat mammary carcinoma induced by 1-methyl-1-nitrosourea. Breast Cancer Res Treat.

[B23] Rooney WD, Springer CS (1991). The molecular environment of intracellular sodium: ^23^Na NMR relaxation. NMR in Biomedicine.

[B24] Liebling MS, Gupta RK (1987). A comparison of intracellular sodium ion concentrations in neoplastic and nonneoplastic human tissue using ^23^Na NMR spectroscopy. Ann NY Acad Sci.

[B25] Barbiero G, Duranti F, Bonelli G, Amenta JS, Baccino FM (1995). Intracellular ionic variations in the apoptotic death of L cells by inhibitors of cell cycle progression. Experimental Cell Res.

[B26] Zs.-Nagy I, Lustyik V, Lukacs G, Zs.-Nagy V, Balazs G (1983). Correlation of malignancy with intracellular Na:K ratio in human thyroid tumors. Cancer Res.

[B27] Elledge SJ (1996). Cell Cycle Checkpoints: preventing an identity crisis. Science.

[B28] King KL, Cidlowski JA (1995). Cell cycle and apoptosis: common pathways to life and death. J Cell Biochem.

[B29] Bruno R, Hille D, Riva A, Vivier N, ten Bokkel Huinnink WW, van Oosterom AT, Kaye SB, Verweij J, Fossella FV, Valero V, Rigas R, Seidman AD, Chevallier B, Fumoleau P, Burris HA, Ravdin PM, Sheiner LB (1998). Population pharmacokinetics/pharmacodynamics of docetaxel in phase II studies of patients with cancer. J Clin Oncol.

[B30] Gorczyca W, Gong J, Darzynkiewicz Z (1993). Detection of DNA strand breaks in Individual Apoptotic cells by the *in situ *terminal deoxynucleotidyl transferase and nick translation assays. Cancer Research.

[B31] Harootunian AT, Kao JPY, Eckert BK, Tsien RY (1991). Fluorescence ratio imaging of cytosolic free Na in individual fibroblasts and lymphocytes. J Biol Chem.

[B32] Charriaut-Marlangue C, Margaill I, Represa A, Popovici T, Plotkine M, Ben-Ari Y (1996). Apoptosis and necrosis after reversible focal ischemia: an in situ DNA fragmentation analysis. J Cereb Blood Flow Metab.

[B33] Summers RM, Joseph PM, Renshaw PF, Kundel HL (1988). Dextran-magnetite: a contrast agent for sodium-23 MRI. Magn Reson Med.

[B34] Hashimoto T, Ikehira H, Fukuda H, Yamaura A, Watanabe O, Tateno Y, Tanaka R, Simon HE (1991). In vivo sodium-23 MRI in brain tumors: evaluation of preliminary clinical experience. Am J Physiol Imaging.

[B35] Turski PA, Houston LW, Perman WH, Hald JK, Turski D, Strother CM, Sackett JF (1987). Experimental and human brain neoplasms: detection with in vivo sodium MR imaging. Radiology.

[B36] Winkler SS, Thomasson DM, Sherwood K, Perman WH (1989). Regional T2 and sodium concentration estimates in the normal human brain by sodium-23 MR imaging at 1.5 T. J Comput Assist Tomogr.

